# Individuals Diagnosed with Binge-Eating Disorder Have DNA Hypomethylated Sites in Genes of the Metabolic System: A Pilot Study

**DOI:** 10.3390/nu13051413

**Published:** 2021-04-22

**Authors:** Mariana Lizbeth Rodríguez-López, José Jaime Martínez-Magaña, David Ruiz-Ramos, Ana Rosa García, Laura Gonzalez, Carlos Alfonso Tovilla-Zarate, Emmanuel Sarmiento, Isela Esther Juárez-Rojop, Humberto Nicolini, Thelma Beatriz Gonzalez-Castro, Alma Delia Genis-Mendoza

**Affiliations:** 1 Genomics of Psychiatric and Neurodegenerative Diseases Laboratory, National Institute of Genomic Medicine (INMEGEN), Mexico City 01090, Mexico; marianalrl_1802@hotmail.com (M.L.R.-L.); jimy.10.06@gmail.com (J.J.M.-M.); 2 Biomedical Postgraduate Program, Academic Division of Health Sciences, Juárez Autonomous University of Tabasco, Villahermosa 86000, Mexico; daruiz_914@hotmail.com (D.R.-R.); iselajuarezrojop@hotmail.com (I.E.J.-R.); 3 Children’s Psychiatric Hospital “Dr. Juan N. Navarro”, Mexico City 01090, Mexico; anarosagarciab@gmail.com (A.R.G.); emmanuelsarmientoh@hotmail.com (E.S.); 4 National Institute of Psychiatry “Dr. Ramón de la Fuente Muñiz”, Mexico City 01090, Mexico; macias@imp.edu.mx; 5 Genomics Laboratory, Comalcalco Multidisciplinary Academic Division, Juárez Autonomous University of Tabasco, Villahermosa 86000, Mexico; alfonso_tovillaz@yahoo.com.mx; 6 Genomics Laboratory, Academic Division Jalpa de Mendez, Juárez Autonomous University of Tabasco, Jalpa de Mendez 86200, Mexico; thelma.glez.castro@gmail.com

**Keywords:** eating disorders, binge-eating disorder, bulimia nervosa, DNA methylation, AMPK

## Abstract

Binge-eating disorder, recently accepted as a diagnostic category, is differentiated from bulimia nervosa in that the former shows the presence of binge-eating episodes and the absence of compensatory behavior. Epigenetics is a conjunct of mechanisms (like DNA methylation) that regulate gene expression, which are dependent on environmental changes. Analysis of DNA methylation in eating disorders shows that it is reduced. The present study aimed to analyze the genome-wide DNA methylation differences between individuals diagnosed with BED and BN. A total of 46 individuals were analyzed using the Infinium Methylation EPIC array. We found 11 differentially methylated sites between BED- and BN-diagnosed individuals, with genome-wide significance. Most of the associations were found in genes related to metabolic processes (*ST3GAL4, PRKAG2,* and *FRK*), which are hypomethylated genes in BED. Cg04781532, located in the body of the *PRKAG2* gene (protein kinase AMP-activated non-catalytic subunit gamma 2), was hypomethylated in individuals with BED. Agonists of PRKAG2, which is the subunit of AMPK (AMP-activated protein kinase), are proposed to treat obesity, BED, and BN. The present study contributes important insights into the effect that BED could have on *PRKAG2* activation.

## 1. Introduction

Bulimia nervosa (BN) and binge-eating disorder (BED) are classified as eating disorders (EDs), which are mental disorders characterized by an alteration in eating behaviors [1,2]. These disorders are present worldwide, with a prevalence ranging from 1.0% to 2.0% [3,4,5,6]. BN and BED are highly related but differ in terms of restrictive behavior. Individuals diagnosed with BN have a fear of gaining weight in combination with recurrent binge eating episodes (i.e., consumption of large amounts of food in short periods of time, with a loss of control), followed by compensatory behaviors [7,8]. Meanwhile, individuals diagnosed with BED have binge eating episodes, but these episodes are not followed by compensatory behaviors [1,8]. BED has recently been recognized as a separate disorder, while it was previously included in eating disorders not otherwise specified. Studies of this disorder are still missing [9,10]. Despite the differences between BN and BED, some authors have proposed that BED could be a severe eating disorder. This increased severity is related to a higher presence of psychiatric metabolic comorbidities [10,11,12,13,14,15,16,17], the ability to differentiate between these disorders is essential for health providers, as it allows them to provide the correct treatment. Later, eating disorders were considered adult-onset disorders, but recent studies have reported that the early-onset of these disorders is correlated with a higher severity [18,19,20,21].

BN and BED are considered complex traits, since their etiology is the effect of the accumulation of different biological, social, and environmental factors [22,23]. In this sense, epigenetics is the processes that influence the expression of genes, without changing the gene structure [22,24]. Epigenetics has been proposed as the link between environmental changes and gene expression. It is the process by which an organism changes its phenotype in response to the environment [25]. The environment could be as simple as the cell microenvironment or as complex as metabolic changes, drug use, or stressful stimuli [26]. The most studied epigenetic change in humans is the dysregulation of the DNA methylation profile [22,27,28]. DNA methylation is found in the 5′ position of the cytosine, and it is necessary for the repression of gene expression [29,30,31,32,33]. However, the study of methylation in eating disorders is still lacking. The main studies have focused on candidate regions, with few studies on genome-wide DNA methylation scanning [24]. DNA methylation occurs in the 5′ position of cytosine, and guanine is in the next position. This cytosine is known as the CpG site, and if it is methylated, it is also called a methylated site [23]. To date and as far as we know, there has not been any epigenetic study on individuals diagnosed with BED [23]. The hypomethylation of the promoter of the atrial natriuretic peptide and dopamine transporter in individuals diagnosed with BN has been reported [23,34,35]. Additionally, another study of ED-diagnosed individuals demonstrated hypermethylation on the oxytocin receptor, which was negatively correlated with body mass index (BMI) [36], pointing to a relationship between the effect of ED on BMI and epigenetic changes. Nevertheless, the epigenetics of ED is still an emerging field.

Several studies are exploring epigenetic factors, but no studies have focused on the differences between disorders. As mentioned, BN and BED are differentiated in terms of compensatory behaviors. Nevertheless, it is not known whether the presence of these compensatory behaviors is enough to promote epigenetic changes. In this sense, the present study aims to compare the genome-wide DNA methylation profiles of individuals diagnosed with BN and BED. We hypothesized that the lack of compensatory behaviors could promote epigenetic changes between BN and BED. As far as we know, this is the first study to explore DNA methylation data in Mexican adolescents diagnosed with EDs.

## 2. Materials and Methods

### 2.1. Study Design

This was a cross-sectional study, with a sample-based design.

### 2.2. Sample Population

In this analysis, we included a total of 46 individuals, with 25 diagnosed with BN and 21 with BED. The individuals were recruited from the area of external consultation of the Hospital Psiquiátrico Infantil Juan N Navarro. The inclusion criteria included children or adolescent (age between 8–17 years), enrolled between 2016–2018, diagnosed with BED or BN only, non-tobacco users, and neither medical nor illegal drugs users. All evaluations were performed by psychiatrists specializing in eating disorders. The recruitment was performed by means of psychiatric treatment. The psychiatrist invited the children or adolescents to participate by speaking with their parents, with a previous explanation that not accepting to participate would not impact their treatment in any way. Besides, no monetary compensation was delivered. Once the aims of the study were exposed, the children/adolescents signed an informed assent, and their parents signed an informed consent. The evaluation of eating patterns was performed using the Spanish version of the Questionnaire on Eating and Weight Patterns-5 (QEWPR-5) and the Eating Attitude Test-26 [37,38]. The present work was approved by the research and ethics committee of the Hospital Psiquiátrico Infantil Juan N Navarro (Number = II3/01/0913) and the Instituto Nacional de Medicina Genómica (Number = 06/2018/I).

### 2.3. Anthropometric and Clinical Characteristics

The age, gender, scholarship, and parents/grandparents’ country of birth of the participants were collected using a structured questionnaire of all the individuals. The anthropometric measurements collected included the weight and height, as previously reported [39]. The body mass index (BMI) was determined according to the obesity task force criteria. One of the limitations of the use of BMI on children and adolescents is that this parameter could be influenced by the development of the child. In order to conduct a comparison of the participants’ BMI, we transformed this value into z-score (z-BMI) values, according to previous reports [40,41]. Screening of binge-eating behavior was performed with the QEWPR-5 and EAT-26, and the diagnostic confirmation was performed by the children’s specialized psychiatrist. QEWPR-5 is a structured questionnaire that allows for the screening of binge-eating disorders based on the DSM-5 criteria. Meanwhile, the EAT-26 is a structured questionnaire that allows for the diagnosis of bulimia nervosa.

The mean age of the participants was 13.91 (Table 1). Of the total 45 adolescents, 12 were males, and 34 were females. Non-individuals diagnosed with BED had compensatory behavior. The mean z-score value for body mass index (BMI) in the total sample was 1.25. Thus, based on the 2007 WHO growth chart reference for school-age children and adolescents, 12 of the participants were overweight, and 19 were obese [40]. The individuals with BED had a higher BMI, compared to the BN individuals (t = −4.02, *p*-value = 2.24 × 10^−4^), as evaluated by the Student’s *t*-test. As expected, none of the individuals diagnosed with BED had compensatory behavior.

### 2.4. DNA Extraction and Microarray DNA Methylation

Once the diagnostic was confirmed, the informed assent/consent was signed, and only if the adolescents accepted to donate the sample, a tube with blood and EDTA (anticlotting agent) was collected by venipuncture. Whole blood samples were collected, and the DNA was extracted using the Gentra Puregene Blood Kit (Qiagen, Germantown, MD, USA), according to the manufacturer’s protocol. The DNA was converted using bisulfite with the Zymo research kit (Zymo, Irvine, CA, USA). The bisulfite-converted DNA was hybridized to the Illumina Infinium Methylation EPIC microarray beadchip (Illumina, San Diego, CA, USA), following the manufacturer’s microarray protocol. The fluorescence intensity was scanned using iScan, and it was transformed into *idat* files using the GenomeStudio software (Illumina, USA). Quality control was also applied using the *ChAMP* package [42], according to previously published algorithms. Briefly, we removed the probes using (i) *p*-value detection (higher than 0.01); (ii) less than 3 beads in less than 5% of the samples; (iii) all non-CpGs sites; (iv) SNP-associated probes; (v) sexual chromosome-associated probes; and (vi) multi-hit probes. Samples with a ratio higher than 0.1 were also removed. Normalization was performed using the beta-mixture quantile normalization method, batch effect removal (slide, array, gender, and age), and adjustment by blood cell proportions were performed, following the algorithm implemented in *ChAMP* [43].

### 2.5. Differential Methylated Sites Analysis

For these analyses, we used the beta values from the quality control file of the *ChAMP* package, and a comparison of the groups was performed using the linear models of the *limma* package [44]. We compared BN with BED and also analyzed BMI (with the z-scores of BMI as the numerical variable). We considered a value statistically significant if the *p*-values were lower than 5 × 10^−8^ (genome-wide significance). We annotated the sites to their gene location (1st Exon, 3′UTR, 5′UTR, Body, Exon Bound, TSS1500, and TSS200) based on the EPIC array manifest. Based on the sample size, we performed a power analysis using the *pwrEWAS* package [45]. Expecting a difference of betas (delta of Beta) of 0.03, we reached a statistical power of 41.0%.

## 3. Results

### Differentially Methylated Sites in BN and BED

**Figure 1:**
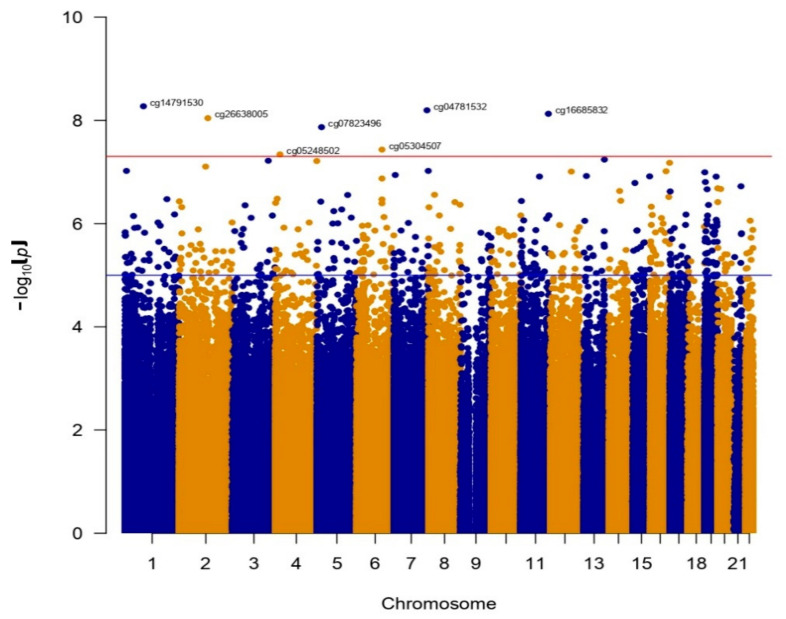
Manhattan plot of the comparison of BED and BN.

In the comparison of bulimia nervosa and binge-eating disorder, we found 11 differentially methylated sites at a genome-wide level (*p*-value < 5 × 10^−8^) (Figure 1).

Of the 11 sites, 4 were intergenic, and 7 were located in gene-coding regions (*CTBS*, *SLITRK3*, *PACRGL*, *FRK*, *PRKAG2*, *ST3GAL4*, and *BANP*) (Table 2). In the gene position, 2 sites were in transcription starting sites, 2 sites were in the gene body, 1 was in the first exon, and 1 was in the untranslated region.

Only three sites were CpG islands, 6 were in Open Sea, 1 was in the shelf, and 1 was on the shore. Of the 11 CpG sites, 9 were hypomethylated in individuals diagnosed with BED. The cg00059161 and cg05248502 were hypermethylated in BED-diagnosed individuals. The higher difference between the groups was found in the cg05304507 associated to the promoter of *FRK*. Additionally, cg05304507 was hypomethylated in individuals diagnosed with BED. In the analysis of differentially methylated sites with BMI, we did not find any CpG site that reached a genome-wide association (*p*-value < 5 × 10^−8^).

## 4. Discussion

Binge-eating disorder (BED) and bulimia nervosa (BN) are differentiated compensatory behaviors in individuals diagnosed with BED. During a binge eating episode, individuals tend to eat fat-rich foods and, as a consequence, have a higher caloric intake [46,47,48,49]. The higher caloric intake has a direct impact in promoting a higher BMI in individuals diagnosed with BED (also seen in this study), and as a consequence, this promotes a higher metabolic disturbance (like adiposity increase) [50,51,52]. Some studies using genetic scoring and Mendelian randomization studies have proposed that epigenetic changes are a consequence of this metabolic disturbance [53,54,55]. One gene that could be influenced by this metabolic disturbance is *PRKAG2. PRKAG2* encodes the γ2-subunit isoform of the AMP-activated protein kinase (AMPK), which is a cellular energy sensor and modulates energy homeostasis [56,57,58]. AMPK is a heterotrimeric serine-threonine kinase that senses cellular energetics, and the activated form triggers the catabolic process and represses anabolic biosynthesis [59,60]. Beyond the metabolic effect, AMPK is essential for the orexigenic effect of ghrelin [61,62,63]. Constitutively, the expression of an active form of AMPK, by recombinant adenoviral expression, acutely increases mice food intake and body weight, suggesting the central role of AMPK in the regulation of eating behavior [64,65]. Furthermore, the chronic expression of *PRKAG2* promotes hyperphagia and obesity [66]. In homology, we can see that individuals diagnosed with BED had hypomethylated *PRKAG2*, suggesting a higher expression of the gene, compared to individuals diagnosed with BN. Nevertheless, the effect of the activation of the former depends on the neuron population [67]. The present result could have a high relevance in terms of the design and use of drugs to activate the AMPK pathway used in the treatment of BED, BN, obesity, and diabetes mellitus type II [68,69,70,71].

Another gene with hypomethylated CpG sites in individuals diagnosed with BED, and not in those diagnosed with BN, is *ST3GAL4.* ST3GAL4, ST3 beta-galactoside alpha-2,3-sialyltransferase 4, is one of the six enzymes that catalyzes Sia-2,3Gal linkages at the ends of glycoproteins. A deficiency of this enzyme in mice is concomitant with depression- and anxiety-like behavior [72,73]. Such behaviors could be important in the modulation of the mood of individuals diagnosed with BED, principally because depression and anxiety are the more common comorbidities found in individuals with this condition.

The gene that showed the most differences was *FRK* (fyn-related kinase). FRK is a 59KDa tyrosine kinase protein that belongs to the Src family [74,75]. The former has recently been reported to play a crucial role in diabetes induction and the increase of proinflammatory signals [76,77,78]. The activation of *FRK* induces cytotoxic signals to the beta pancreatic cells in response to several cytokines or beta cell toxins (like streptozotocin) [79,80], thus promoting the induction of diabetes. Our results showed a hypomethylation of *FRK* in BED, compared to BN, suggesting an overexpression of the gene, which could lead to a higher induction of cytotoxic signals. This change in methylation could point to a higher risk of metabolic dysfunction in individuals diagnosed with BED, compared to those diagnosed with BN.

Even when we found interesting associations in the effect that BED could have on epigenomes, compared to BN, we noticed some limitations of our work. The main one is the sample size, which imitates the statistical power of our associations. Another limitation is associated with the lack of biochemical and food intake data, principally during the binge eating episodes. These data could help us to better characterize the epigenetic changes due to environmental sources. Another point to mention is that we did not find any associations with BMI, which may be because this construct cannot be perfectly measured in individuals diagnosed with ED. This limitation could pose some issues, as individuals diagnosed with ED are generally more worried about other weight constructs than they are about BMI [81]. Nevertheless, further analysis of the phenotype and a larger sample are required to replicate our findings.

## 5. Conclusions

Individuals diagnosed with BED showed hypomethylation in genes of the metabolic system, and those diagnosed with BN did not. The present result could be important in connection with the use of agonists or activators of AMPK in the treatment of BED, BN, or obesity.

## Figures and Tables

**Table 1:** Sample description.

	BN (*n* = 25)	BED (*n* = 21)	Total (*n* = 46)
Gender			
Female (*n*, %)	22 (88.00)	12 (57.14)	34 (73.91)
Male (*n*, %)	3 (12.00)	9 (42.86)	12 (26.09)
Age (s.d)	13.76 (1.56)	14.10 (1.51)	13.91 (1.53)
Body Mass Index			
BMI z-score (s.d)	0.87 (0.74)	1.69 (0.65)	1.25 (0.81)
Normal weight (*n*, %)	14 (56.00)	1 (4.76)	15 (32.61)
Overweight (*n*, %)	5 (20.00)	7 (33.33)	12 (26.09)
Obese (*n*, %)	6 (24.00)	13 (61.90)	19 (41.30)
Eating behavior			
Compensatory (*n*, %)	25 (100.0)	0 (0.00)	25 (47.83)
Binge eating (*n*, %)	17 (68.00)	21 (100.00)	38 (82.61)

Notes. BN = Bulimia Nervosa, BED = binge-eating disorder, s.d = standard deviation.

**Table 2:** Differentially methylated sites in bulimia nervosa and binge-eating disorder.

Position ^1^	CpG Site	LogFC ^2^	*p*-Value	BED Avg	BN Avg	Gene	Gene Loc ^3^	CGI ^4^
1:85040857	cg14791530	−0.0499	5.3305 × 10^−^^9^	0.8150	0.8550	*CTBS*	TSS1500	Shore
2:131090049	cg26638005	−0.0509	9.0552 × 10^−^^9^	0.3135	0.3644		IGR	OpenSea
3:164907027	cg00059161	0.0216	6.0427 × 10^−^^8^	0.7929	0.7713	*SLITRK3*	Body	OpenSea
4:20702180	cg05248502	0.0211	4.5662 × 10^−^^8^	0.0737	0.0526	*PACRGL*	1st Exon	Island
4:189258348	cg22740817	−0.0138	6.1212 × 10^−^^8^	0.9427	0.9565		IGR ^5^	OpenSea
5:20305935	cg07823496	−0.0386	1.3534 × 10^−^^8^	0.9039	0.9424		IGR	Island
6:116381966	cg05304507	−0.0765	3.6910 × 10^−^^8^	0.7500	0.8265	*FRK*	TSS200	OpenSea
7:151565722	cg04781532	−0.0190	6.3694 × 10^−^^9^	0.9376	0.9566	*PRKAG2*	Body	OpenSea
11:126284163	cg16685832	−0.0282	7.4533 × 10^−^^9^	0.8750	0.9032	*ST3GAL4*	3′-UTR	Shelf
13:114318347	cg21211187	−0.0115	5.7405 × 10^−^^8^	0.9501	0.9616		IGR	OpenSea
16:88110197	cg10838260	−0.0245	6.6465 × 10^−^^8^	0.8966	0.9211	*BANP*	Body	Island

Notes: ^1^ Human genome position of the CpG site (GRCh37/hg19). ^2^ LogFC = logarithm of fold change. ^3^ Gene Loc = location of the CpG site relative to the coding gene. ^4^ CGI = CpG island. ^5^ IGR = Intergenic region.

## Data Availability

The data presented in this study are available in the Supplementary material. Additional data are available on request from the corresponding author, which were omitted due to privacy and ethical issues.
